# Fiber Forming Capability of Binary and Ternary Compositions in the Polymer System: Bacterial Cellulose–Polycaprolactone–Polylactic Acid

**DOI:** 10.3390/polym11071148

**Published:** 2019-07-04

**Authors:** Mehmet Onur Aydogdu, Esra Altun, Jubair Ahmed, Oguzhan Gunduz, Mohan Edirisinghe

**Affiliations:** 1 Centre for Nanotechnology & Biomaterials Research, Department of Metallurgical and Materials Engineering, Faculty of Technology, Marmara University, Goztepe Campus, 34722 Istanbul, Turkey; 2 Department of Mechanical Engineering, University College London, Torrington Place, London WC1E 7JE, UK; 3 Department of Metallurgical and Materials Engineering, Faculty of Technology, Marmara University, Goztepe Campus, 34722 Istanbul, Turkey

**Keywords:** polycaprolactone, polylactic acid, bacterial cellulose, polymers, fibers

## Abstract

Bacterial Cellulose (BC) has over recent decades shown great versatility in wound healing dressings, but is difficult to spin fibers with at high concentrations. An investigation into the preparation of bandage-like fibrous meshes is carried out to determine the optimal blend of polycaprolactone (PCL) and polylactic acid (PLA) as a suitable carrier for BC. Using a simple centrifugal spinning setup, polymer blends of PCL, PLA and BC are investigated as a ternary system to determine the most suitable composition with a focus on achieving maximal BC concentration. It is found that BC content in the fibers above 10 wt % reduced product yield. By creating blends of PLA-PCL fibers, we can create a more suitable system in terms of yield and mechanical properties. The fibrous samples are examined for yield, fiber morphology using scanning electron microscopy, mechanical properties using tensile testing and chemical characteristics using Fourier-transform infrared spectroscopy. A fibrous scaffold with > 30 wt % BC was produced with enhanced mechanical properties owing to the blending of PLA and PCL.

## 1. Introduction

Skin is the largest organ in the human body, and the most effective natural barrier against dehydration, temperature loss, pathological microorganisms and thus plays a vital role in maintaining homeostasis [1]. Therefore, whenever the skin integrity is compromised due to various factors such as diseases, burns and traumas, consequences can lead to huge loss of function in normal health. Epidermal healing by reepithelialization is the natural self-regeneration mechanism and is considered to be the most reliable wound recovery method [2]. However, this process can deteriorate due to disease and other pathological factors or simply prove to be inadequate against other serious wounds caused by skin cancer or third degree burns. According to reports from the World Health Organization (WHO), more than 300,000 deaths annually have been reported relating to skin burns and wounds [3]. Therefore, wound dressings become increasingly important and crucial for the support of wound healing mechanisms in the body. Thus, there is a growing pressure to produce wound care materials that will facilitate the natural course of recovery.

Even though the basic concept of a wound dressing has evolved via the exploration of a broad range of materials since ancient times, management of wounds still remains a significant medical challenge and a huge burden to the economy despite recent progress [4,5]. In England, 184 million pounds were spent in 2012 on the development of wound dressings and this number is only growing with increasing population [6]. The production of affordable and effective wound dressings is a significant challenge for scientists and healthcare professionals. There are certain requirements in designing an effective wound dressing which is compatible with the complex mechanisms of biological regeneration whilst still retaining a low cost. Previous studies have focused on producing biocompatible mats using natural and synthetic polymer blends, tailored into scaffolds consisting of fibrous structures using advanced engineering approaches [4,7,8,9]. Despite the promising results, certain improvements can be made to the wound dressing model using different materials and improving the current production methods. Finding the best synergy between material combinations and tailoring those scaffolds with a proper production method can only reduce costs and can lead to its widespread use.

Centrifugal spinning-based fiber forming methods demonstrate high production rates and easy solution feeding which allows for the large-scale manufacture of fibrous constructs [10,11,12]. These systems show great potential for industrial upscale due to their ability to rapidly form constructs and not be limited by charge-interferences as can be seen with other fiber forming techniques [13]. Polylactic acid (PLA) is a medically safe synthetic polymer that has already been used for various tissue engineering applications and has proven to have a high biological affinity [14]. However, the brittle characteristic of PLA can be a limitation for many tissue engineering applications. On the other hand, Polycaprolactone (PCL), is one of the most commonly used polymers in tissue engineering due to its advantageous properties such as elasticity, biodegradability, biocompatibility and appropriate mechanical properties [15,16,17,18].Blends of PLA and PCL have been previously reported and have been proven to be suitable polymer blends [19,20].

Another material, Bacterial cellulose (BC), which complements the hydrophobicity of synthetic polymers with its unique features, is a low cost, biocompatible polymer with excellent water absorption capabilities and mechanical properties which makes it a highly suitable additive for wound dressings [21,22,23]. Compared to cellulose derived naturally from plants and algae, BC possesses a thinner microfibrillar structure which leads to better mechanical properties such as in its tensile strength and Young’s Modulus [24,25,26,27]. However, BC is extremely difficult to process and produce with current engineering procedures. Due to the production rate of BC being limited by static bacterial cultures, its yield is very low and thus cannot currently be exploited commercially. Because of its various advantageous properties, BC in wound dressings is highly desirable as it can prevent infection, provide a suitable niche for cells of the wound response and ultimately creates a much improved healing result [4].

In this study, using binary and ternary combinations of PLA, PCL and BC, a gyration-based fiber forming method is used to produce bandage-like scaffolds. These composite scaffolds are assessed on their yield, mechanical, physical and chemical properties in order to discover the ideal combination of these materials for bandage applications, all whilst keeping yield as high as possible.

## 2. Materials and Methods

### 2.1. Materials

BC was obtained from Centre for Nanotechnology & Biomaterials Research, Marmara University (Istanbul Turkey). PLA (MW = 110 × 10^3^ g mol^−1^), PCL (MW = 80 × 10^3^ g mol^−1^) and Chloroform (CAS: 67-66-3) was purchased from Sigma-Aldrich (Gillingham, UK).

### 2.2. Preparation of Blend Solutions

Firstly, BC membranes were cut into small pieces with a lancet and were placed onto a petri dish whilst a napkin was pressed against it for 30 s to soak up the excess water.. Thereafter, samples were placed in a beaker with the appropriate amount of chloroform and subjected to sonication for 1h using a sonifier (Branson SFX550, BRANSON Ultrasonics Corporation, Danbury, CT, USA). The BC was then centrifuged with ethanol to remove any excess water in the BC and was subsequently stored in an airtight vial at ambient temperature (22–24 ℃). 12 wt % PLA and 12 wt % PCL solutions were used and prepared in chloroform which was stirred for 24 h at ambient temperature (22–24 ℃). Subsequently, different BC blend ratios were created (0 to 100) with PLA and PCL to make binary systems at different weight ratios. The polymers were mixed under ambient conditions (22–24 °C). 90 wt. % BC was found to be the limit of PLA-BC and PCL-BC binaries and fibers did not form beyond this composition (Table 1). Thereafter, prepared PLA and PCL solutions were blended at different ratios (10 to 90). Finally, since the main aim of the study was to maximise BC content without compromising the bandage shape and yield, BC concentration within the (PLA-PCL)-BC ternary system was gradually increased (0 to 90). Similar to the afformentioned binaries, 90 wt. % BC was also the limit for PLA-PCL-BC ternary systems and no fibers were collected beyond that. All blend solutions for binary and ternary systems were used without further treatment.

### 2.3. Bandage-Like Scaffold Production

**Figure 1:**
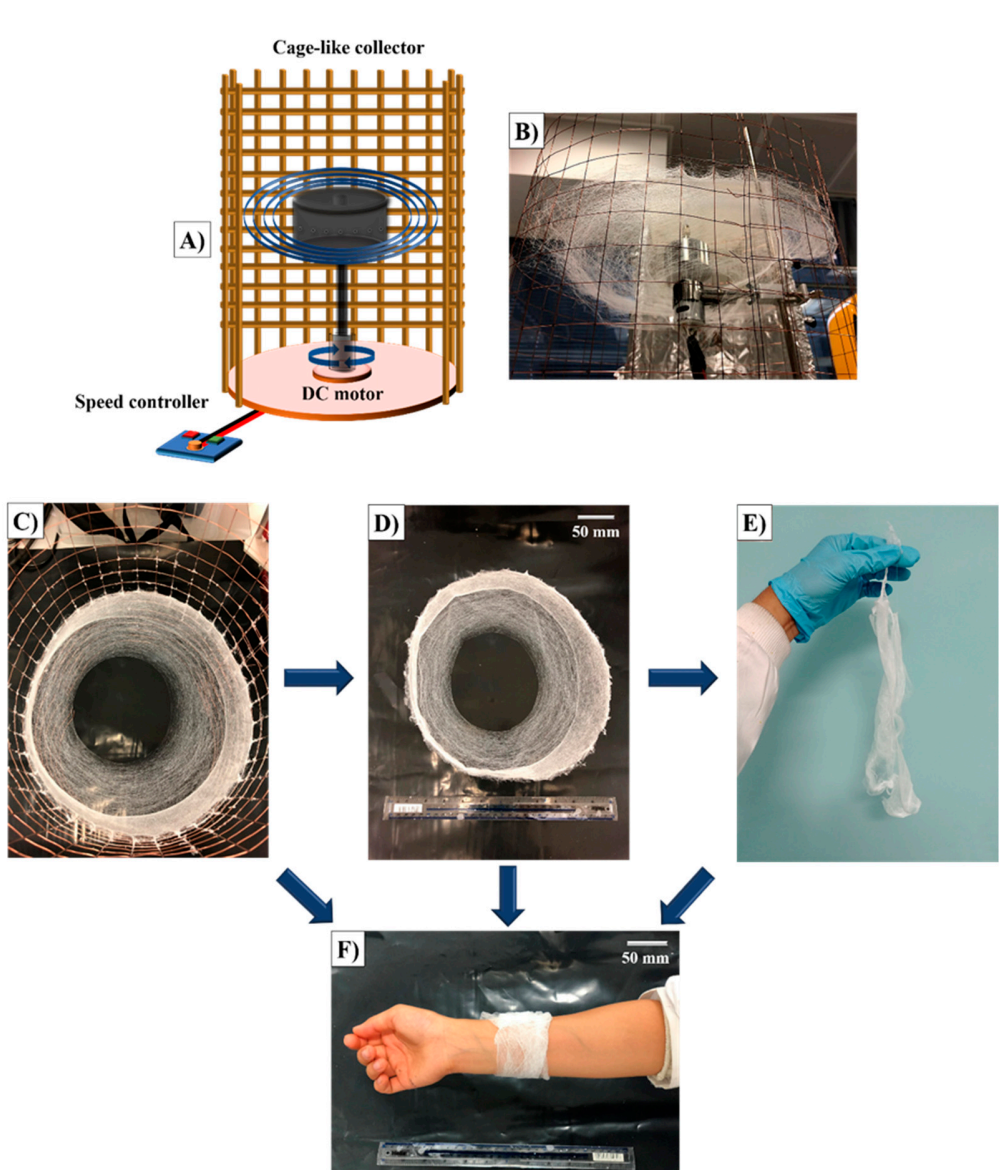
Bandage-like fibrous scaffold production: (**A**) simple centrifugal spinning system, (**B**) the cage collector with deposited fibers, (**C**) bandage-like fibrous scaffold in the collector, (**D**) appearance of the bandage-like sample after collection, (**E**) image of the hand-held sample and (**F**) image of the sample as wrapped around an arm.

Basic centrifugal spinning was used for the production of constructs analyzed in this work (Figure 1A). All experiments were conducted in a rotary aluminum cylindrical vessel (≈60 mm in diameter and ≈35 mm in height) which had 24 circular orifices (0.5 mm in diameter) on its exterior. The bottom of the vessel was connected to a DC motor which provided a maximum rotational speed of 36,000 rpm. For these experiments, the rotational speed was fixed at 36,000 rpm. The high speed rotation of the vessel allows for the formation of a polymer jet and this dries to produce fibrous scaffolds which escape through the orifices to the collector (Figure 1B). All experiments were run for 15 s at 23–24 ℃ ambient temperature and 51%–54% relative humidity. After the spinning process, the cage around the spinneret was carefully removed (Figure 1C) and bandage-like fibrous scaffolds were carefully collected (Figure 1D). These mats could be successfully handled manually (Figure 1E) allowing it to be wrapped around epidermal tissue (Figure 1F).

Following the experiments, a vacuum oven was used to evaporate any residual chloroform that may have remained in the bandage-like fibrous scaffolds. At the end of the experiments, the optimal blending ratio was found by characterization tests and calculating the yield.

### 2.4. Characterization

#### 2.4.1. Physical

Physical characterization tests (viscosity, density, and surface tension) of all the prepared solutions were carried out. A Brookfield DV-III ULTRA viscometer (Brookfield Viscometers Ltd., Harlow, UK) was used to measure the viscosity using a small sample adapter with a volume of 3 mL. A digital tensiometer (K9, Kruss GmbH, Hamburg, Germany) was used to evaluate the surface tension of the solutions via the Wilhelmy’s plate method. All equipment was calibrated before use and all measurements were repeated three times at ambient temperature (22–24 °C) and a relative humidity of 40%–50%.

The following equation was used to determine yield percentage values of the spinning process and all preparations were repeated three times:

Yield % = 100 (Obtained solid product weight (g) / Loaded solution’s weight (g))(1)

#### 2.4.2. Morphological

For visualization of the fibers in bandages, a ZEISS MA EVO 10 scanning electron microscope (SEM, Zeiss, Oberkochen, Germany) was used at an accelerating voltage of 10 kV. Before imaging, samples were sputter coated with gold for 60s using a Quorum SC7620 Mini Sputter Coater. The diameters of the fibers were measured with the image processing program, ImageJ.

#### 2.4.3. Chemical

The bandage samples prepared by spinning were characterized by FTIR for their chemical features, using a JASCO FT/IR-4000 spectrometer. On the instrument, each spectrum was taken at a resolution of 4 cm^−1^, and with a range between 4000–400 cm^−1^.

#### 2.4.4. Mechanical

The thickness of each fibrous scaffold was measured three times at different points using a Mitutoyo, High-Accuracy Digimatic Micrometer (Kawasaki, Japan) before each was tensile tested. Average thickness of each sample was used for the determination of tensile strength. The stress-strain properties of the samples were calculated using tensile extension with a 30 mm gauge length by a uniaxial tensile test machine (INSTRON 4411, MA, USA). Test cycles were performed using a load sensor of 50 N and a loading speed of 5 mm/min in ambient temperature (22–24 ℃). The stress-strain curves were recorded, the maximum tensile strength and Young’s modulus values were obtained. Three samples were measured for each bandage-like fibrous samples allowing for the mean and standard deviation to be calculated.

## 3. Results and Discussion

### 3.1. Yield Calculations

**Figure 2:**
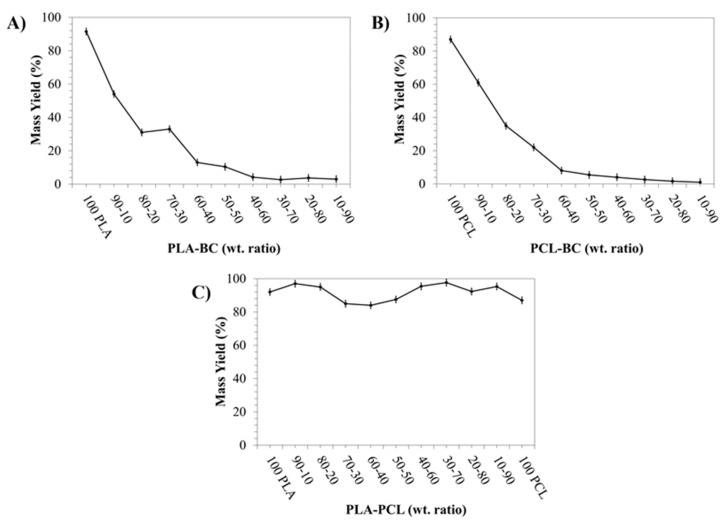
Percentage yield values for binary systems; PLA-BC (**A**), PCL-BC (**B**), and PLA-PCL (**C**) bandage-like fibrous samples.

Centrifugal spinning techniques and its more modern counterparts allow for a high production rate of small diameter fibers [28]. In this study, the goal was to determine the optimal yield of PLA-PCL-BC systems while retaining a bandage like shape and mechanical properties. Thus, there was no attempt to the make the fiber forming stage a variable and state-of-the-art [28]. The relative yields are compared in Figure 2 for binary blends. With regards to the percentage yield of PLA-BC samples (Figure 2A), the yield is 91.5 ± 1.2% for 100% PLA and decreased to 54.0 ± 1.4% at a ratio of 90:10 PLA-BC. This decreased further to 33.0 ± 1.4 % in the 70:30 PLA-BC sample before falling gradually to 3.0 ± 0.4% at the 10:90 PLA-BC ratio. Likewise, 87.0 ± 1.4 % yield of fiber was obtained for the 100% PCL sample after which the yield dropped to 61.0 ± 1.2% for the 90:10 PCL-BC sample (Figure 2B). The yield decreased to 22.0 ± 1.4% in the 70:30 PCL-BC ratio sample before dropping to 1.0 ± 0.4% for the 10:90 PCL-BC sample. On the other hand, the yield of fibers was 97.0 ± 1.5% at the starting point of 90:10 PLA-PCL binary blend system before progressively decreasing to 84.0 ± 1.9% at 60:40 PLA-PCL (Figure 2C). The yield was seen to rise to 87.5 ± 1.8% for the 50:50 PLA-PCL and peaked at 97.6 ± 0.8% at 30:70 PLA-PCL before decreasing to 95.3 ± 1.0% at a ratio of 10:90 PLA-PCL.

**Figure 3:**
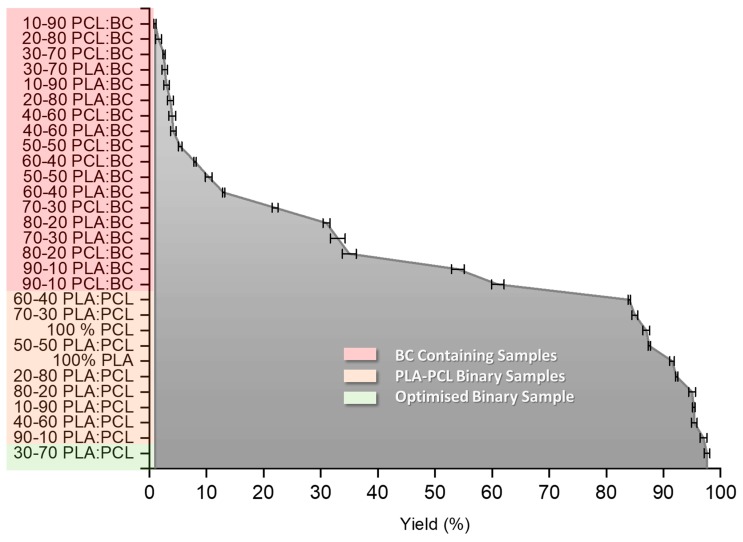
Graph showing all of the tested binary systems and their corresponding yield values.

It is seen from Figure 2A,B that the yield decreases drastically as the ratio of PLA and PCL are altered. It is not fully understood why this occurs but is assumed to be due to the change in polymer chain entanglement, matrix and change in solution characteristics. A BC concentration of 30 wt % was selected as the optimal blend with the PLA-PCL binary system, since otherwise a significant yield drop was observed and the bandage-like shape began to be compromised at higher concentrations of BC. Since the main aim was increasing the BC content without losing the proper bandage shape in the PLA-PCL-BC ternary system, BC concentration within the 30:70 PLA-PCL blend was gradually increased to find the best ratio in terms of yield and bandage integrity. Due to its highest yield, 30:70 PLA-PCL solution was chosen and blended with BC to make the most optimal (PLA-PCL)-BC ternary system. Even though fibers were formed at higher that 70:30 (PLA-PCL)-BC ratios, the yield was drastically reduced and a loss in bandage shape integrity was observed. From Figure 3, we can see the relationship between the PLA-PCL, PLA-BC and PCL-BC binary systems and the production yield. We see again that the highest yield was achieved by blending PLA and PCL at a ratio of 30:70. It is observed in the orange region how the yield falls off at different PLA-PCL ratios. The red region indicates all of the PLA-BC and PCL-BC blends fibers, we can see that the addition of BC at any concentration causes a reduction in yield.

### 3.2. Morphological Features of the Scaffolds

**Figure 4:**
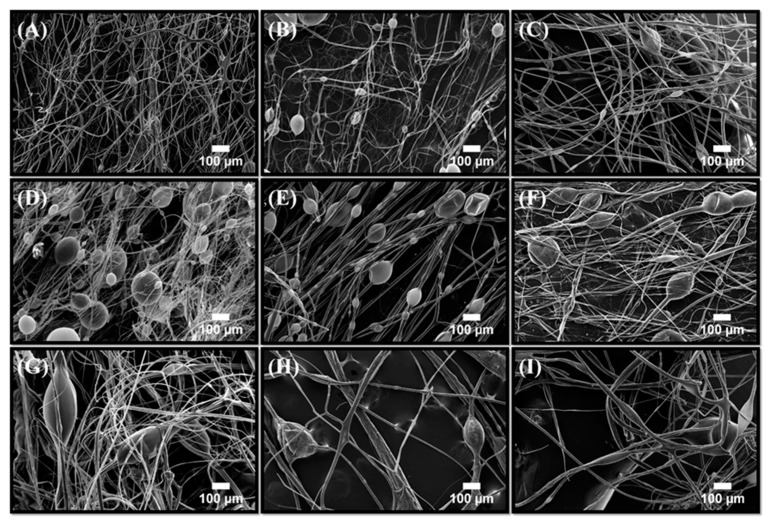
Scanning electron microscopy images of PLA-BC fibers produced; (**A**) 90:10 PLA-BC, (**B**) 80:20 PLA-BC, (**C**) 70:30 PLA-BC, (**D**) 60:40 PLA-BC, (**E**) 50:50 PLA-BC, (**F**) 40:60 PLA-BC, (**G**) 30:70 PLA-BC, (**H**) 20:80 PLA-BC and (**I**) 10:90 PLA-BC.

Fiber mats consisting of binary (PLA-BC, PCL-BC, PLA-PCL) and ternary ((PLA-PCL)-BC) microstructures were characterized using scanning electron microscopy. Figure 4A–I shows the SEM micrographs of the mentioned fiber samples. At the lowest BC concentration, unaligned fibers were observed with a low bead frequency. As the BC concentration increases, the prevalence of these bead-like structures increased.

**Figure 5:**
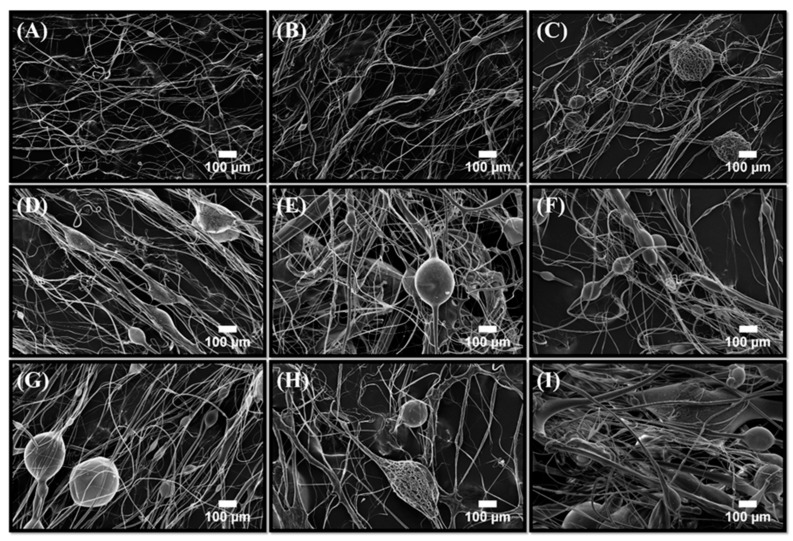
Scanning electron microscopy images of the PCL-BC fibers produced with; (**A**) 90:10 PCL-BC, (**B**) 80:20 PCL-BC, (**C**) 70:30 PCL-BC, (**D**) 60:40 PCL-BC, (**E**) 50:50 PCL-BC, (**F**) 40:60 PCL-BC, (**G**) 30:70 PCL-BC, (**H**) 20:80 and (**I**) 10:90 PCL-BC.

Similar results were also observed with PCL-BC systems as seen in Figure 5A–I, which shows the images of the fibers from a starting ratio of 90:10 to 10:90. Unaligned fibers with extremely low bead frequency were observed at the highest PCL ratio and by gradually decreasing the polymer ratio and increasing BC within the composite structures caused the bead density to increase. Previous studies have reported that increasing the BC concentration within the PCL-BC composites lead to an increase in bead occurrence [7]. Furthermore, incorporation of BC into other polymeric materials also resulted in similar outcomes [16,29]. Therefore, the increase in the number of beads in the fibers can be explained by simply the presence of BC. Relating to yield values, SEM images showed a lower fiber count when yield was low, thus possibly indicating that the lower yield may be due to the bead evolution while jetting polymer fiber. Adding BC to the samples decreased yield, fiber frequency and efficiency of the production as well as compromising the mechanical integrity of the constructs.

**Figure 6:**
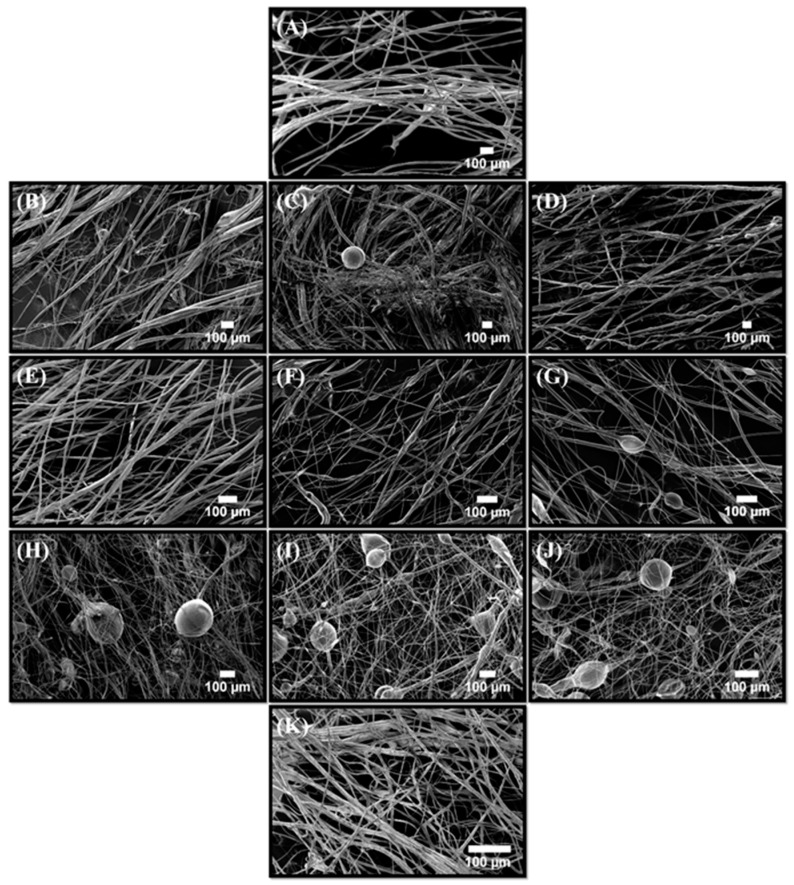
Scanning electron micrographs of the PLA-PCL fibers produced with; (**A**) 100:0 PLA-PCL, (**B**) 90:10 PLA-PCL, (**C**) 80:20 PLA-PCL, (**D**) 70:30 PLA-PCL, (**E**) 60:40 PLA-PCL, (**F**) 50:50 PLA-PCL, (**G**) 40:60 PLA-PCL, (**H**) 30:70 PLA-PCL, (**I**) 20:80 PLA-PCL, (**J**) 10:90 PLA-PCL and (**K**) 0:100 PLA-PCL.

Morphological changes in the PLA-PCL binary combinations are also revealed and discussed in Figure 6A–K. It shows 100 % PLA fibers followed by an increasing blend of PCL. Results indicated that the bead frequency increased in parallel with the PCL concentration. This is a well-known characteristic for PLA-PCL composite structures, PCL will tend to bring more particle and bead-like structures when forming a fiber matrix with another polymer. A previous study also demonstrated the same interactions between PLA and PCL and reported similar bead forming mechanics along with variations of polymer concentrations [30].

The diameter of fibers is an important characteristic as it can determine many features that come with the available surface area to volume ratio [31]. The increased surface area can lead to greater functionality which can provide advantages to the constructs such as better cell proliferation and higher mechanical properties [32,33]. Reduction of the average fiber diameter of the scaffold material during the manufacturing process is highly desirable for tissue engineering and wound healing applications [34]. Here, viscosity, surface tension, and solvent evaporation rate of the polymer solution determine the fiber diameters of the final product [35,36]. Final fiber diameter can be tailored by factors that also influence yield of the products, such as rotational speed. Therefore, each of these parameters and the physical properties highlight the importance of a centrifugal spinning setup that allows equilibrium between solvent evaporation and mass transfer of the polymeric materials. In addition, pressure in the vessel and infusion of feed can be used as processing controls and thus can also significantly improve fiber alignment [28].

**Figure 7:**
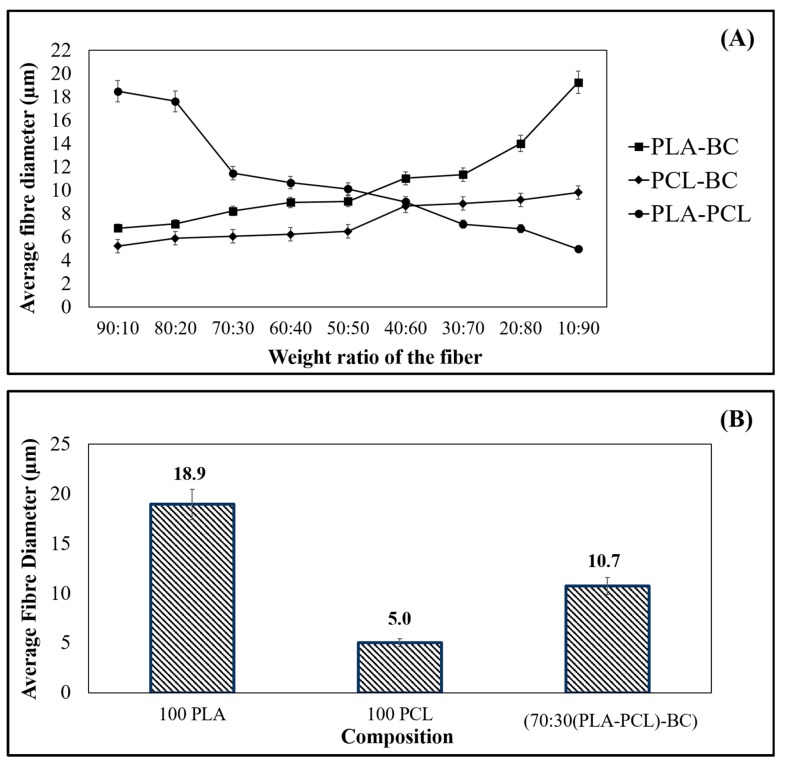
Graphical representation of the average fiber diameters for each material combination used: (**A**) Binaries of (PLA-BC, PCL-BC, and PLA-PCL), (**B**) Virgin PLA, PCL and ternary composite (70:30 (PLA-PCL)-BC).

Figure 7A shows the average fiber diameter values for the binary combinations (PLA-BC, PCL-BC, and PLA-PCL) investigated in this study and Figure 7B shows the average fiber diameters of primary materials (PLA and PCL) and the optimal ternary sample (70:30 (PLA-PCL)-BC). Diameters of 100% PLA and 100% PCL fibers recorded an average of 18 µm and 5 µm respectively while the binary combinations fluctuated, ranging from 5.0 µm to 18.5 µm. The results here were comparable with other studies using similar materials and equipment [37,38]. This is believed to be related to the aerodynamics in the spinning vessel (Figure 1), providing better evaporation and allowing the fiber jets to travel more and freely without being limited within closed chambers, eventually decreasing the fiber diameter and producing more promising fiber microstructures in the materials. Centrifugal spinning-based methods are devoid of an applied electric field and allow polymers without a charge to be spun with a large production rate [7,39].

In this study, the diameters of the PLA-BC composite fibers varied between 6 µm and 19 µm while the PCL-BC fibers had diameters between 5 and 9 µm. Fiber diameters for each binary group and the ternary product (70:30 (PLA-PCL)-BC) increased as the BC content increased, even when the polymer ratios changed in each sample produced. This can be related to the polymer weights, viscosities and surface tension of the solutions since the production and environmental parameters were kept constant. As the viscosity and surface tension increases, fiber diameters also increased for samples containing BC, this trend has also been previously reported [16]. The addition of BC within each composite leads to an increase in viscosity and a slight increase in surface tension (Table 2), this resulted in the formation of thicker fibers whenever the BC content increased (Figure 7A). Furthermore, it is well established that the higher viscosity and surface tension also have important effects on the production of thicker polymeric fibers [35]. Reduction of the PLA polymer concentration, which had a higher viscosity than PCL, resulted in a decrease in observed fiber diameters for both binary and ternary systems. Previous studies reporting the results of polymeric fibers generated by other centrifugal spinning related scenarios also resulted in similar outcomes in terms of solution effects on diameters [40,41,42].

### 3.3. Chemical Characterization of the Scaffolds

**Figure 8:**
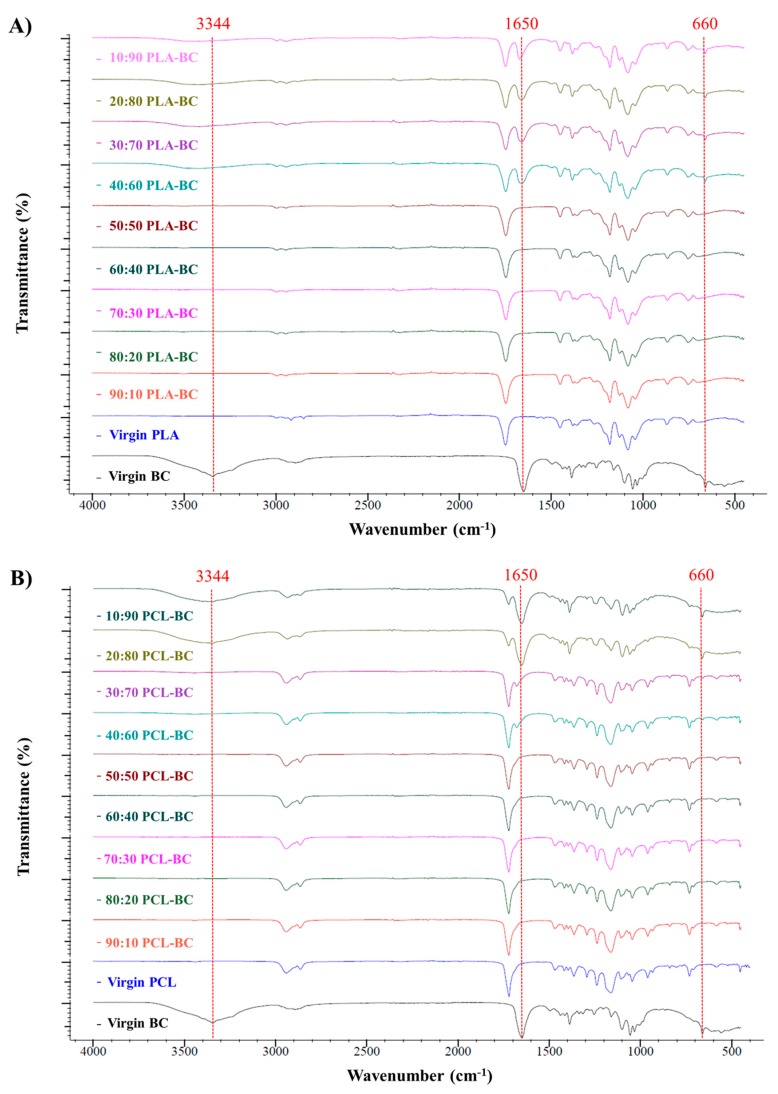
FTIR spectra of (**A**) PLA-BC fiber blends compared to Virgin PLA and BC, (**B**) PCL-BC fiber blends compared to Virgin PLA and BC.

**Figure 9:**
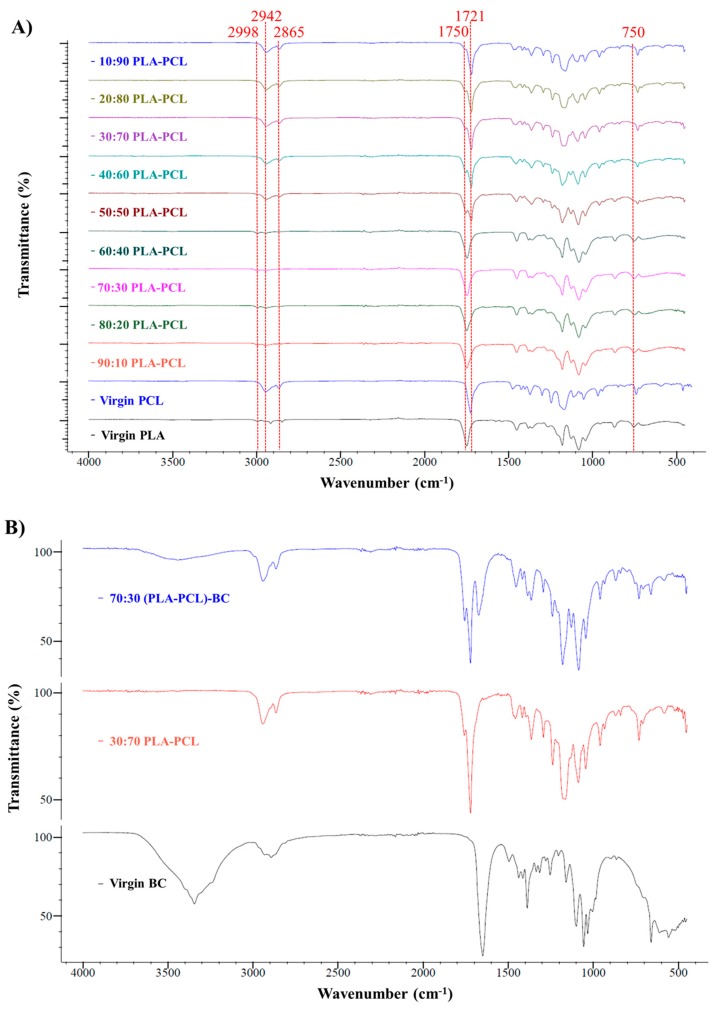
FTIR spectra for of (**A**) PLA-PCL fiber blends compared to virgin PLA and BC, (**B**) Optimized ternary sample compared with optimized binary and virgin BC.

FTIR spectroscopy was used to confirm the interactions between the components in the spun fibers. Figure 8 presents the FTIR spectra of virgin PLA, BC and composite PLA-BC fibers (Figure 8A), virgin PCL, BC, and composite PCL-BC fibers (Figure 8B), while Figure 9 shows composite PLA-PCL fibers (Figure 9A) and the optimized ternary (PLA-PCL)-BC sample (Figure 9B). Characteristic bands for PLA, PCL, and BC are observed in all relevant samples. Observed bands at 1750 cm^−1^ and in the range of 1452-755 cm^−1^ were attributed to PLA in Figure 8A [43]. Also, characteristic absorption bands of BC at the wavenumbers of 3344, 1650 and 660 cm^−1^ became more visible in the samples of 40:60 PLA-BC to 10:90 PLA-BC [44]. In Figure 8B, the characteristic absorption band of BC at the wavenumbers of 3344 cm^−1^ became more discernible in the samples of 20:80 PCL-BC and 10:90 PCL-BC. Additionally, characteristic peaks between 1650 and 660 cm^−1^ appeared in the samples between 40:60 PCL-BC and 10:90 PCL-BC. All other peaks at 2942, 2864, 1720, and 731 cm^−1^ were related to the presence of PCL [45]. For PLA-PCL blends, PLA peaks at 2998, 1750 and 755 cm^−1^ lost intensity to PCL at the peaks of 2942, 2865, 1721 and 731 cm^−1^ as the ratio of PCL increased in the blend (Figure 9A). All polymers showed their characteristic peaks in the optimized ternary blend sample (70:30 (PLA-PCL)-BC) and polymer miscibility with each other was evidenced in this spectrum. As shown in Figure 9B, peaks at 3344, 1650 and 660 cm^−1^ are attributed to BC, peaks at 2942, 2864, 1720 and 731 cm^−1^ are ascribed to PCL and peaks at 1749 and 755 cm^−1^ belong to PLA polymer [46].

### 3.4. Mechanical Properties of the Scaffolds

**Figure 10:**
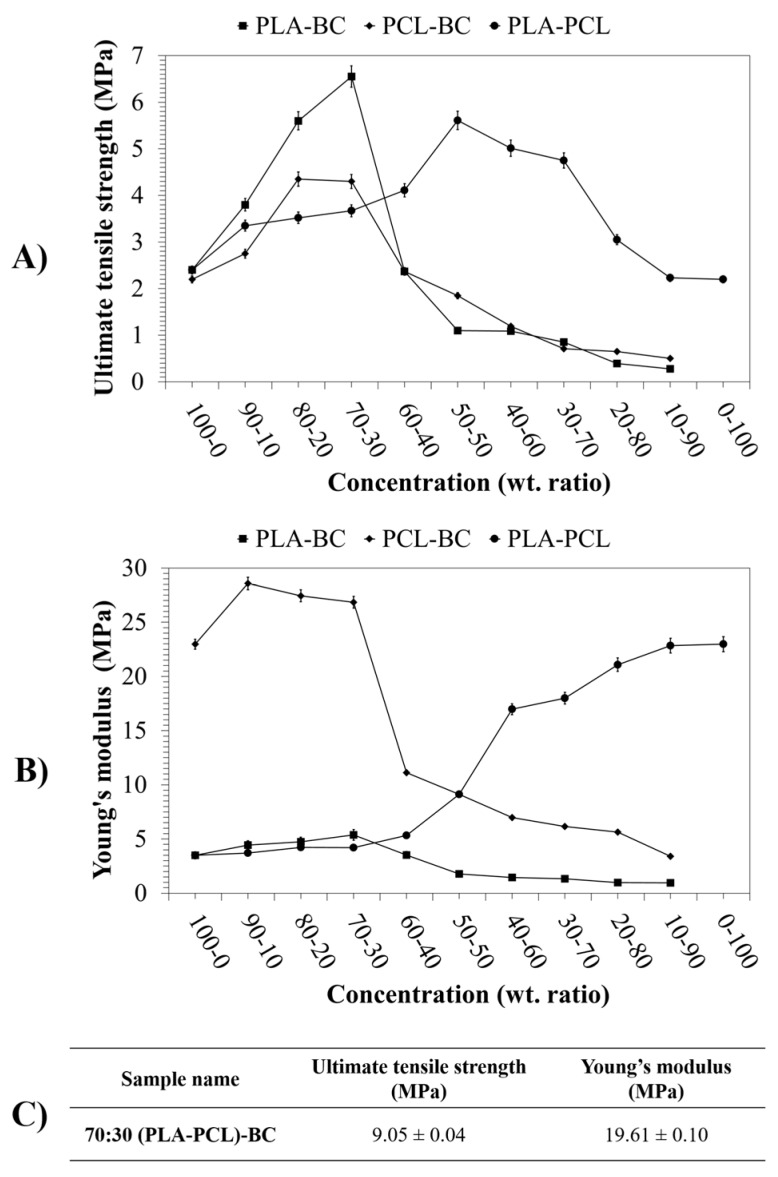
Tensile results of the samples: (**A**) Ultimate tensile strength of the binary systems, (**B**) Young’s modulus of the binary systems, and (**C**) Ultimate tensile strength and Young’s modulus of the ternary sample.

Strength is a crucial requirement for proper wound dressings which is expected to be compatible with their surroundings in terms of mechanical features and should also be able to provide the desired biomechanical protection [47]. Tensile testing of binary systems and the ternary sample was thus carried out to determine the effect of polymer and BC composition on the mechanical properties of such constructs. The ultimate tensile strength and Young’s modulus values of each binary system and the ternary systems are displayed in Figure 10.

Ultimate tensile strength values of the PLA-PCL binary systems fluctuated between 2.2 MPa and 5.6 MPa while the Young’s modulus values varied between 3.5 MPa and 22.3MPa. As reported in previous literature, PLA-PCL blends are very significant examples for the incorporation of two polymeric phases in terms of mechanical properties [48]. PLA is known for its high ultimate tensile strength but suffers from low toughness, which can be compensated for by PCL’s significant elongation at break; eventually displaying enhanced mechanical properties in their composite products by merging beneficial characteristics [49,50,51]. PLA-PCL blend systems displayed good elongation and tensile properties at 50:50 ratios in this study.

It is observed that in all cases, the increased ratio of BC in the composite fibers leads to an increase in tensile strength up to 30 wt %, beyond this point a fall in the tensile strength is observed. This fall also coincides with the loss in yield that is observed in the production process and elucidates the difficulty in further increasing BC content. The stiffness of PLA composite systems increases with BC content. However, an inverse pattern is seen in PCL systems where the stiffness actually decreases. This decrease can be explained by the mechanical compatibility of PLA-PCL and the role of relative slippage between the two polymers. The highly elastic nature of the PCL combined with the PLA matrix and the bonding forces between the polymers prevented fiber stretching, which lead to enhanced mechanical properties of the composites [48,52,53,54].

Samples with BC showed similar results in both PLA and PCL composites. Young’s modulus values decreased from 23.0 MPa to 3.4 MPa for PCL binary systems while binary systems of PLA ranged between 1.0 MPa 3.5 MPa as the BC concentration increased. On the other hand, the ultimate tensile strength values of PLA-BC binary systems ranged between 0.3 MPa and 6.6 MPa, PCL-BC showed a range between 0.5 MPa and 4.4 MPa.

The incorporation of BC in the composite leads to an increase in ultimate tensile strength in both scenarios as reported in previous studies. This can be explained by the ultra-fine network structure of BC, its extremely significant physico-chemical properties, and its natively high tensile strength [54]. In the polymer blends produced, it is expected that the addition of BC caused interactions between the BC microfibrils and the polymers chains which lead to enhanced mechanical properties [55]. Additionally, the ternary sample produced in this study had a tensile strength of 9.1 MPa and a stiffness of 19.6 MPa. This optimized ternary composite possessed the elasticity of PCL with the high mechanical strength of PLA and BC, whilst retaining high yields and structural integrity. However, after a critical point, yield drastically fell off to undesirable levels with increases in BC concentration, especially after 30 wt. %. This significant diminishing of the yield and ultimate tensile strength is explained by the addition of bacterial cellulose to the blends which effects the fiber forming mechanism of the spinning system, hindering both quality and quantity of the fibers being produced in a single run.

## 4. Conclusions

This comprehensive investigation demonstrates characteristics of blends of important biomedical polymers for use in healthcare related applications. Using a simple centrifugal spinning setup, we were able to thoroughly investigate numerous combinations of PLA, PCL and BC to determine the optimal composition for yield, morphology and bacterial cellulose content in bandage-like fibrous scaffolds. The results presented here show that it is possible to achieve as high as 30 wt % BC in fibrous scaffolds. Compared to previous studies, the incorporation of the BC increased three times. Furthermore, a ratio of 70:30 (PLA-PCL) was established as the optimal binary polymer blend as it gave the best compromise between yield, morphology and mechanical properties. Scaffolds with over 30 wt.% BC were also produced and studied, but due to the dramatic fall in yield, the bandage-like shape was compromised. Further optimization to more advanced spinning techniques such as pressurized gyration and its sister processes may allow for the possibility of higher loadings of BC and other constituent materials. This work shows a promising outlook for the future of composite polymer blends and shows the effects of both binary and ternary systems. The work presented here shows a facile methodology to produce bandage-like mats for wound healing and as such, these mats will be suitable as a subject for future work.

## Figures and Tables

**Table 1:** Blend ratios of binary system solutions.

PLA-BC Ratio (wt. ratio)	PCL-BC Ratio (wt. ratio)	PLA-PCL Ratio (wt. ratio)
90:10	90:10	90:10
80:20	80:20	80:20
70:30	70:30	70:30
60:40	60:40	60:40
50:50	50:50	50:50
40:60	40:60	40:60
30:70	30:70	30:70
20:80	20:80	20:80
10:90	10:90	10:90

**Table 2:** Solution properties of binary systems and the ternary sample.

Ratio	PLA-BC		PCL-BC		PLA-PCL	
	Viscosity (Pa s)	Surface Tension (mN m^−1^)	Viscosity (Pa s)	Surface Tension (mN m^−1^)	Viscosity (Pa s)	Surface Tension (mN m^−1^)
100:0	363 ± 1	52.8 ± 0.1	258 ± 1	38.4 ± 0.1	363 ± 1	52.8 ± 0.1
90:10	368 ± 2	53.9 ± 0.1	269 ± 2	39.2 ± 0.2	358 ± 1	51.3 ± 0.2
80:20	375 ± 2	55.2 ± 0.2	288 ± 3	41.4 ± 0.3	351 ± 2	50.8 ± 0.2
70:30	403 ± 1	57.2 ± 0.3	301 ± 1	43.3 ± 0.1	346 ± 1	49.5 ± 0.1
60:40	445 ± 01	59.5 ± 0.3	342 ± 1	46.4 ± 0.1	331 ± 2	48.2 ± 0.2
50:50	462 ± 2	60.1 ± 0.2	371 ± 2	48.8 ± 0.2	321 ± 2	47.1 ± 0.1
40:60	481 ± 1	61.5 ± 0.3	432 ± 2	51.3 ± 0.3	314 ± 1	44.2 ± 0.1
30:70	503 ± 1	63.3 ± 0.1	476 ± 3	54.2 ± 0.3	301 ± 3	43.8 ± 0.1
20:80	521 ± 2	65.5 ± 0.2	525 ± 3	57.6 ± 0.4	295 ± 2	41.4 ± 0.2
10:90	544 ± 3	67.2 ± 0.1	573 ± 2	59.4 ± 0.1	284 ± 2	39.2 ± 0.2
0:100	N/A	N/A	N/A	N/A	258 ± 1	38.4 ± 0.1
**Ternary Sample:** 70:30 (PLA-PCL)-BC **Viscosity:** 359 ± 2 Pa s **Surface Tension:** 46.1 ± 0.3 mN m^−1^						
